# “We’re all learning together”: exploring peer educator engagement in Recovery Colleges through a participatory research approach

**DOI:** 10.3389/fpsyt.2025.1601408

**Published:** 2025-07-07

**Authors:** Betsabeh Parsa, Angela Towle, Sue Macdonald

**Affiliations:** ^1^ Research Coordinator, Faculty of Medicine, University of British Columbia, Vancouver, BC, Canada; ^2^ Associate Professor Emeritus, Department of Medicine, University of British Columbia, Vancouver, BC, Canada; ^3^ Lead, Consumer Involvement and Initiatives, Vancouver Coastal Health Authority, Vancouver, BC, Canada

**Keywords:** Recovery College, peer educators, peer support, Participatory Action Research, lived experience

## Abstract

**Introduction and purpose:**

Recovery Colleges offer a community-based, recovery-oriented approach that promotes mental health and personal growth through co-produced, peer-led courses. Despite their growth in Canada, limited research examines factors influencing peer educators’ sustained engagement—an essential aspect of program sustainability. This study addresses this gap by identifying key factors and developing best practices to support peer educators in Recovery Colleges and enhance retention and well-being.

**Methods:**

This study employed a mixed-methods Participatory Action Research (PAR) approach, engaging peer educators as co-researchers. A Committee of seven local peer educators (five remained actively involved) co-designed tools and interpreted findings as the advisory peer educators. All Canadian Recovery Colleges were invited to participate. Data were collected from peer educators and program organizers via an online survey and virtual interviews (n=32, across nine provinces). Qualitative data were analyzed using thematic analysis, with coding refined through an iterative process.

**Results:**

We identified five themes for sustaining peer educator engagement: Inclusivity, Connectedness, Adaptability, Empowerment, and Implementation Factors. Practical recommendations emerged for recruitment, training, and workplace support. The findings emphasize the need for inclusive, adaptable, and empowering environments to sustain peer educator engagement in Recovery Colleges.

**Discussion:**

Centring peer educator experiences is critical to upholding Recovery Colleges’ values and creating inclusive, meaningful learning environments that promote personal and community growth. The participatory nature of the research highlighted the unique insights of our advisory peer educators and echoed the Recovery College principles of promoting recovery and building on individual strengths.

## 1 Introduction

Rates of mental health and substance use disorders, particularly in adolescents, have increased over the past decade (1–4). This trend has been deepened by the COVID-19 pandemic, which has further elevated the risk of mental health disorders, particularly among those with pre-existing conditions (5–8). For example, between 2012 and 2022, the 12-month prevalence of generalized anxiety disorder among Canadians aged 15 and older increased from 2.6% to 5.2%, while major depressive episodes rose from 4.7% to 7.6%, with youth exhibiting even greater increases (9). Despite these rising mental health burdens in Canada and globally, counseling and psychotherapy services remain largely out of reach due to long wait times, high costs, and accessibility barriers (9, 10). Approximately one-third of individuals with a recent mental health disorder did not receive adequate care, with unmet needs for counseling or therapy surpassing those for medication or information (9). These challenges underscore the urgent need for accessible, community-based, and preventative mental health interventions (7, 8, 11).

Among these emerging approaches, Recovery Colleges offer a promising model for bridging service gaps and promoting personal recovery (12). Personal recovery, defined as an individual’s transformation of attitudes, values, emotions, goals, abilities, and roles to live a fulfilling and meaningful life despite illness (13), has become a central focus in mental health policy worldwide (14). This process is often framed by the CHIME framework, which emphasizes five key components: Connectedness, Hope and optimism, Identity, Meaning and purpose, and Empowerment (15). Consequently, mental health strategies increasingly aim to enhance individuals’ ability to manage their lives, build social connections, cultivate purpose, develop essential life and work skills, and expand educational opportunities (16). Achieving this requires a comprehensive, holistic approach to mental health care that includes a multi-level commitment to implementing community-based, recovery-oriented interventions.

Community-based, recovery-oriented service delivery and peer-led interventions can improve mental health service access and contribute to well-being (17). These approaches emphasize supportive community environments, empowering individuals through shared experiences, reducing social isolation, and fostering resilience (10). The Recovery College model exemplifies this approach, offering evidence-based education on mental health, recovery, and well-being for all community members, regardless of mental health or substance use challenges (12).

Originating in London in 2009, Recovery Colleges have since expanded to over 220 locations worldwide, emphasizing an adult education approach that fosters personal growth, skill development, and empowerment (16, 18). The defining principles of Recovery Colleges include educational approaches, co-production, co-facilitation, co-learning, recovery-focused and strengths-based perspectives, as well as evolution, community integration, and inclusivity (19). These principles emphasize respect, mutuality, and a person-centered approach to create inclusive learning environments (20, 21). Similarly, the RECOLLECT study identifies recovery, adult education, co-production, connectedness, and a community focus as key components of Recovery Colleges (19, 22). Additionally, an international study by King and Meddings (23) identified features such as the recovery approach, valuing lived experience, co-production, education, and inclusivity as consistently present within Recovery Colleges.

Studies associate Recovery College attendance with positive outcomes, such as improved well-being, achievement of recovery goals, and enhanced self-management skills, with potential benefits for healthcare practices (18, 24). A key component of Recovery Colleges is co-production, where participants’ lived experiences are valued as expertise. In this collaborative model, courses are co-designed and co-delivered by peer educators (individuals with lived and living experience of mental illness) and practitioners (24, 25). Co-production involves a collaborative process among diverse stakeholders to identify needs, develop programming, implement interventions, and conduct evaluations (26–28). This inclusive approach challenges traditional power hierarchies, empowers participants, and centers lived experience as a form of expertise.

The role of peer educators is vital. Peer educators, individuals with lived experience of mental health challenges, play a central role in Recovery Colleges by promoting empowerment, mutual learning, and challenging traditional power dynamics (29, 30). Their participation brings authenticity to the educational environment, models recovery, and fosters inclusive, stigma-reducing spaces (31). Co-creation is a cornerstone of the Recovery College model, serving as a guiding principle for all program activities (27). Community integration, alongside empowerment, is essential to fostering transformative change in mental health recovery (32). As such, peer-led approaches are essential to recovery-oriented mental health care, enabling those with lived experience to shape services and drive sustainable, community-rooted change (32).

Despite their pivotal role, peer educators remain underrepresented in the academic literature. While existing studies often highlight the experiences of students and clinical staff, limited research focuses exclusively on the perspectives of peer educators who co-produce and deliver Recovery College programming. To our knowledge, no prior study has explored peer educator experiences as a pathway to improving program design and outcomes. This study aims to address that gap by examining the facilitators and barriers to peer educator engagement in Recovery College programming within the Canadian context. Specifically, the study pursued two objectives:

1. Identify key facilitators and barriers to peer educator engagement in the co-production and delivery of Recovery College courses in Canada.
2. Co-develop actionable strategies to sustain peer educator involvement and enhance program effectiveness.

The resulting findings inform the creation of a practical, community-informed toolkit designed to support future Recovery College initiatives and strengthen recovery-oriented practices across mental health services.

## 2 Materials and methods

### 2.1 Context of the study

In Canada, Recovery Colleges are primarily operated by the Canadian Mental Health Association (CMHA), with peer educators and program organizers at their core. Peer educators are individuals with lived and living experience of mental health or substance use challenges who support others by co-creating and delivering recovery-based courses. Program organizers in this study are administrative staff who also actively participate in the co-creation and delivery of courses. They oversee Recovery College operations and integrate peer-led initiatives into broader mental health services. Alongside peer educators and participants, clinicians occasionally contribute to course co-design, forming a third stakeholder group in the Recovery College framework.

As of 2023, there were 30 Recovery Colleges across nine Canadian provinces (Alberta, British Columbia, Manitoba, New Brunswick, Nova Scotia, Ontario, Prince Edward Island, Quebec, and Saskatchewan), offering diverse programming under various names to support mental health recovery education efforts nationwide. All Canadian Recovery Colleges were invited to participate in the study.

### 2.2 Community-based participatory design

This study is part of a broader mixed-methods Participatory Action Research (PAR) initiative aimed at co-developing tools and strategies to strengthen the design and delivery of a new local Recovery College program. PAR is a collaborative research approach that centers experiential knowledge to address problems rooted in unequal and harmful social systems while co-creating transformative alternatives (33). The broader PAR project involves collaboration between researchers, service providers, and individuals with lived and living experience of mental health or substance use challenges involved with the local Recovery College program. Its primary goals were to explore community-informed approaches to recovery education, promote inclusive service design, and generate evidence to support peer-led programming. Peer educators were engaged not only as participants but as research partners throughout all phases of the research, contributing to design, data interpretation, and knowledge mobilization.

Guided by institutional and published recommendations (34, 35), the co-creative and participatory learning approach emphasizes the perspectives of peer educators to enhance the project’s relevance and impact. Specifically, a group of peer educators, who will be referred to as advisory peer educators, participated as an advisory group for the research. Recruitment for these advisory peer educators began through targeted community outreach within local Recovery College programs. Recruitment for the advisory peer educators began through targeted community outreach within local Recovery College programs. Eligible individuals were invited to join if they had lived experience with mental health or substance use challenges, were currently or previously employed as peer educators, demonstrated interest in collaborative research, and were available to participate in meetings. Recruitment methods included email invitations through service providers, informational sessions at Recovery Colleges, and snowball sampling via peer networks.

In January 2023, seven advisory peer educators were recruited to form the Recovery College Project Peer Advisory Committee, which guided the participatory research process. Committee members were selected through a combination of voluntary participation and purposive sampling following outreach to the local Recovery College programs. Of the seven peer educators initially recruited, five remained actively involved until the project’s conclusion. Monthly meetings ensured sustained collaboration, allowing the advisory peer educators to co-design study tools, refine questions, and maintain user relevance. The advisory peer educators received training in social research and knowledge translation and were recognized and compensated for their contributions. A final event celebrated and shared outcomes based on the advisory peer educators’ contributions.

#### 2.2.1 Visual framework: the tree analogy

**Figure 1:**
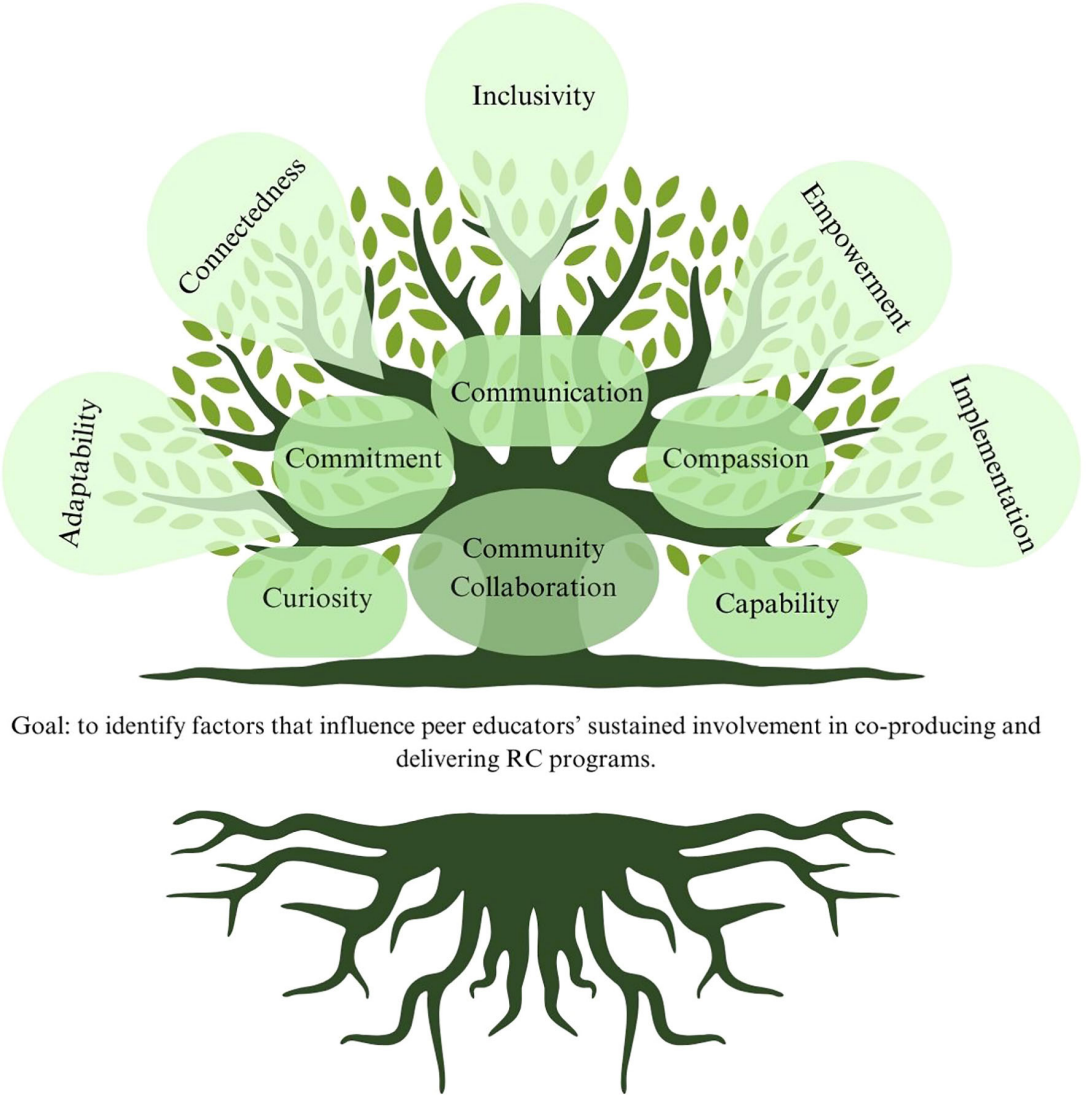
The participatory research process and its outcomes from peer advisors’ perspectives. The image used in this manuscript was created using Canva Pro, following Canva’s Content License Agreement. As per Canva’s licensing terms, Pro users are granted the right to use and publish content generated within the platform, including for academic and commercial purposes, provided that elements used comply with the license type (Free, Pro, or Extended). The image does not violate any intellectual property rights and adheres to Canva’s guidelines for permissible use. For further reference, Canva’s full licensing agreement can be found at: https://www.canva.com/policies/content-license-agreement/.

**Figure 1** developed through this study illustrates the participatory research process and its outcomes from the peer advisor perspective. The tree symbolizes our research process rooted in recovery-oriented and community-based models, nurtured through collaboration and resulting in recommendations. The roots represent foundational principles such as communication, commitment, and compassion, while the fruits represent five themes identified through the study—Inclusivity, Connectedness, Adaptability, Empowerment, and Implementation Factors. The analogy serves as a unifying symbol, emphasizing that these themes are interconnected and contribute collectively to peer advisor engagement.

### 2.3 Research design

The broader study employed a mixed-methods approach, combining an online stakeholder survey and semi-structured interviews to gather comprehensive insights. This report, however, focuses solely on the qualitative data obtained through survey and interviews.

#### 2.3.1 Data collection

We invited two participant groups: peer educators, individuals with lived or living experience of mental health or substance use challenges who used their experiences to contribute to course delivery and content development, and program organizers, who were staff responsible for program administration, coordination, content development, and implementation. Program organizers might draw upon their own lived experience with mental health or substance use challenges, but this was not recognized as central to their role. This structure allowed us to explore shared experiences and broader patterns while still attending to role-specific insights. In smaller Recovery College settings, these roles sometimes overlapped; one participant in our study, for example, served in both capacities.

We co-developed an online survey hosted on Qualtrics, with local advisory peer educators and program organizers validating the questions to ensure relevance. Since building rapport with the study participants was important to us, and that the collection of demographic information has been noted by peers (including our advisory committee) as off-putting (36), these details were optional. Furthermore, we did not end up with enough responses to make the data significant.

Recovery Colleges across Canada were contacted through established networks and community outreach and peer educators were invited to participate via posters, emails, and word-of-mouth referrals. Participation was open to all peer educators and program organizers working with Canadian Recovery Colleges. Study materials, including a poster and survey link, were distributed to initial contacts at each Recovery College for further dissemination.

The study was approved by the University of British Columbia Behavioural Research Ethics Board (ID# H22-03124), and all data were handled confidentially. Informed consent was obtained from all participants. Survey responses were anonymous, though participants could volunteer contact information if interested in receiving an honorarium or participating in a follow-up interview. Survey questions focused on the structure and operations of Recovery Colleges, the roles and experiences of peer educators and program organizers, training and preparation, employment conditions, and perceptions of support and job satisfaction (See **Supplementary Material 1** for a complete list of questions). Only responses to three open-ended survey questions (i.e., relating to participants’ understanding of Recovery Colleges, willingness to recommend the program, and reflections on their experiences as peer educators) were included in the analysis.

Following the survey, we conducted 60-minute semi-structured interviews via a video conferencing platform (Zoom) with interested survey respondents to explore general issues identified in the survey. Interview questions focused on understanding the experiences, challenges, and successes of individuals involved in Recovery Colleges, particularly peer educators (See **Supplementary Material 2** for a complete list of questions). The interviews were conducted by the study research assistant (BP) and interviews were recorded to assist with transcription and review. To show appreciation for their time, all self-identified survey participants and interviewees were offered a small honorarium in the form of a retail store gift card of their choice.

#### 2.3.2 Data interpretation

Qualitative data from both survey responses and interviews were analyzed using an inductive thematic approach (37, 38). The open-ended responses from the survey and interview data were initially coded separately but followed the same systematic process, with responses categorized into themes. Two coders (BP and SM) independently coded the first interview transcription, then met to validate and refine the coding framework before proceeding with the remaining interviews. Survey data were coded using the same framework and later integrated with interview findings to identify overarching themes. Data were organized in Excel, and initial codes were grouped into themes and sub-themes, with relevant quotes selected to illustrate each theme.

To examine the perspectives of both participant groups, peer educators and program organizers, we coded data with role identifiers, allowing us to observe which themes were shared or role-specific. As similar trends emerged across both groups, we ultimately integrated the data to identify overarching themes. However, we remained attentive to the source of each contribution throughout the analysis. This approach allowed us to center the voices of peer educators, while recognizing the complementary perspectives of program organizers.

Themes and sub-themes were finalized through iterative discussions among the advisory peer educators and the research team, ensuring alignment with participants’ perspectives and the study objectives.

## 3 Results

### 3.1 Participant overview

A total of 32 peer educators and program organizers from Recovery Colleges across Canada participated in this study. 20 participants completed the survey, and 12 participated in individual interviews. There was an overall overlap between the survey respondents and interview participants, with all but one interview participant also completing the survey. **Table 1** presents the number of participants from each province.

**Table 1:** Number of participants from each province.

	Province	Number of participants	
		Survey	Interview
1	Alberta	2 (PEs)	2 (PEs)
2	British Columbia	12* (9 PEs; 3 POs)	4 (2 PEs; 2 POs)
3	Manitoba	2 (POs)	2 (POs)
5	Ontario	4 (2 PEs; 2 POs)	3 (1 PEs; 2 POs)
6	Quebec	0	1 (PE)
	Total number of participants	20	12

PE, Peer educator; PO, Program organizer; *6 local PEs, and 1 local POs.

### 3.2 Key themes

As illustrated by the tree analogy we developed through this study (**Figure 1**) and summarized in **Table 2** our qualitative analysis identified five major themes that influence peer educators’ sustained engagement in Recovery Colleges: Inclusivity, Connectedness, Adaptability, Empowerment, and Implementation Factors. Like branches of a tree, these themes are interconnected, collectively influencing peer educators ongoing involvement and offering actionable insights for supporting their roles.

**Table 2:** Themes and sub-themes identified through thematic analysis.

Theme	Sub themes
Inclusivity	a) Embracing diversity b) Promoting equity within the RC team c) Fostering accessibility
Connectedness	a) Communication and collaboration within the RC team b) Collaboration with other RC centres c) Community connection
Organizational Adaptability	a) Fluidity and growth b) Community-centered flexibility
Empowerment	a) Fostering self-growth b) Providing support c) Offering training and education
Implementation Factors	a) Funding b) Roles and responsibilities

The following sections explore identified themes and their associated sub-themes, along with illustrative quotes highlighting key points and providing deeper insight (all names are pseudonyms). Also, practical strategies for enhancing peer educator engagement are summarized in **Table 3**.

**Table 3:** Actionable recommendations to support peer educators by organized by theme.

Theme	Recruitment	Training	Workplace support
Inclusivity	Promote diverse outreach and equitable access to attract underrepresented peer educators.	Provide materials in multiple formats for accessibility (e.g., audio, visual, translations).	Rotate leadership roles to foster equity.
Connectedness	Build local and national networks for sharing resources.	Train peer educators in team-building and communication skills through group training sessions.	Create peer support groups and promote collaboration.
Adaptability	Select peer educators open to uncertainty and responsive to community needs.	Train on flexible facilitation and adult learning styles.	Encourage “failing forward” as part of growth.
Empowerment	Ensure peer educators have a clear personal recovery pathway and are ready to facilitate.	Offer practical and reflective training opportunities.	Provide resources to prevent burnout and encourage growth.
Implementation Factors	Standardize payment and roles to reduce power imbalances.	Train peer educators on operational and facilitation tasks.	Secure funding to support program operations.

#### 3.2.1 Inclusivity

The theme of Inclusivity highlights the importance of embracing diverse perspectives and creating an environment that is accessible and welcoming to all. This theme consists of three sub-themes: embracing diversity, promoting equity within the Recover College team, and fostering accessibility.

Participants emphasized the need to increase the diversity of peer educators as a way to better reflect and engage the diverse communities served by Recovery Colleges. They suggested that expanding the representation of peer educators could encourage broader participation among students from underrepresented groups. This perspective reflects both a recognition of current gaps in representation and a desire to move toward more inclusive practices within the Recovery College model.

Alex, a peer educator, mentioned:

*“I think in the recruiting aspect, diversity and inclusion is really important … there’s no racial diversity on our team … I would say prioritizing diversity in recruitment is important, because representation in staff is so important, regardless of the work that you’re doing. People want to see others they can relate to, connect with, and feel safe with…”*

Equity within Recovery College teams emerged as another critical aspect of Inclusivity. Participants valued non-hierarchical, shared leadership approaches, which ensured all team members, regardless of their role, contributed equally. This approach challenges traditional power dynamics and promotes mutual respect among team members.

As Sally, a peer educator, noted:

*“We’re learning all together. We all have knowledge, we all have the same power, the same place…. In this way, even the trainers don’t pretend they are experts; they’re also learners”.*

Participants also recommended practical strategies to advance Inclusivity, such as rotating responsibilities among peer educators to foster skill-building and shared leadership. Addressing accessibility barriers was another priority. Addressing accessibility barriers was brought up as another priority. Several participants noted that not all peer educators had equal access to digital tools or reliable internet connections, which limited their ability to fully participate in course planning and delivery. Technical barriers, such as unfamiliarity with virtual platforms or lack of support in navigating them, were described as particularly challenging. To mitigate these barriers, suggestions included offering training on digital tools, providing technology support, and using diverse outreach methods to involve individuals who may otherwise be excluded.

#### 3.2.2 Connectedness

The theme of Connectedness emphasizes building strong community ties, fostering open communication, and promoting collaboration within and across Recovery College teams. Participants identified creating a welcoming, non-judgmental environment as essential for helping peer educators and participants feel safe and supported. This theme is reflected in three subthemes: communication and collaboration within the Recovery College team, collaboration with other Recovery College centres, and community connection. As August, a peer educator, explained:

*“I think a lot of people are attracted to peer support at Recovery Colleges because they crave connection. They’re craving community and they don’t know where to find it, or they do know where to find it, but those communities have not been welcoming to them. [Our centre] really strives to be as non-judgmental and as safe as we can be for people to just show up as they are. And I think people respond really well to that…”*

Transparent and authentic communication was also seen as crucial for cultivating trust within Recovery College teams and the broader community. Participants described feeling respected when they could be open about their identities and experiences, reinforcing a culture of acceptance and mutual trust. August, the peer educator shared,

*“I think people are really open to being open and just being like ‘this is who I am’… I can just show up, and people will respect me … as a person, and therefore they respect me as a facilitator”.*

Regular consultation with peer educators to understand their needs, conducting needs assessments, and addressing requests promptly, practices recommended to be led by program organizers, were highlighted as strategies for maintaining strong relationships and ensuring Recovery Colleges remain relevant and responsive.

To support peer educators, participants recommended establishing peer support structures, such as creating peer educator support groups to encourage shared learning and mutual support. Pairing peer educators in courses was suggested to foster collaboration and provide opportunities for debriefing after sessions.

Broader collaboration with other Recovery Colleges was another key aspect of Connectedness. Participants advocated for province-wide or national meetings to share knowledge and best practices. This collaboration could also include sharing course materials across Recovery Colleges to strengthen ties and expand learning opportunities on a larger scale.

#### 3.2.3 Organizational adaptability

Organizational Adaptability was identified as a key theme highlighting the importance of a Recovery College’s ability to remain flexible and responsive to the evolving needs of both the community and peer educators. Participants emphasized that adaptability was not just expected of individual educators, but required at the institutional level, through policies, programming, and leadership practices. This theme consists of two subthemes: embracing fluidity and growth, which refers to peer educators’ openness to evolving roles and responsibilities, and adopting community-centered flexibility, which describes how Recovery Colleges as institutions can adjust their operations and offerings based on the needs and input of the communities they serve.

Participants emphasized the importance of an open mindset toward new ideas, uncertainty, and change, viewing adaptability as essential to Recovery College success. Key suggestions for fostering a growth-oriented culture included encouraging peer educators to share new ideas and embracing uncertainty and evolution within Recovery Colleges. Ruth, a program organizer, illustrated this approach:

*“…You’re [going to] try things; they might not work out so [well]; then, you just fix it and move on. So, we call that failing forward in our team. Because there are so many things that are uncertain, we don’t know how something is [going to] work …. Try what you think is the best of all the ideas and just see how it goes, and then just keep evolving. I think you get into trouble when you just stay stagnant, go. …Everything we do is failing forward!”*

The ability to learn from setbacks was regarded as a crucial aspect of growth, as participants noted that progress may be gradual and requires patience. This mindset of “failing forward” reinforces the importance of learning from experiences and adapting as necessary to improve future outcomes.

Tailoring Recovery College programs to fit the specific needs of the community emerged as a crucial aspect of adaptability. Participants described peer educators as community representatives and as such expressed a preference for the co-design of fewer, high-quality courses that are better aligned with the unique needs of each community they serve. They felt that this approach would allow for deeper engagement, more meaningful content, and greater relevance to participants. Additionally, adapting courses from other Recovery Colleges to better suit local contexts was seen as valuable. By involving peer educators in this adaptation process, Recovery Colleges can ensure programs remain relevant and resonate with the specific communities they serve.

#### 3.2.4 Empowerment

Empowerment emerged as a key theme, capturing participants’ insights on the importance of empowering peer educators within Recovery Colleges. This theme is reflected in three subthemes: providing support, fostering self-growth, and offering training and education.

Participants emphasized that offering emotional, educational, and professional support to peer educators is critical for their empowerment. The emotionally demanding nature of peer support work, which often involves drawing from lived experiences, was highlighted as a significant challenge. Kevin, a program organizer, illustrated this by saying:

*“Within peer values, one of the things that’s missed oftentimes is that someone who is bringing their lived experience into a space and has to work from that standpoint doesn’t get to turn it off at the end of the day … and I don’t think there’s an appreciation for the weight of what that is. I don’t think there’s an appreciation for if people have had challenging experiences within systems and they’re coming back to work in that system because they want to see it change. The impact of being in that environment and engaging with people is not [acknowledged] nor compensated. And that’s a very, very challenging thing. And I think there’s a high rate of burnout for individuals because of stuff like that”.*

To address these challenges, participants suggested wellness plans as an essential tool for preventing burnout and suggested ensuring that peer educators have “fully recovered” before taking on intensive work. As personal recovery is a self-defined process, this topic may be discussed during the interview process and incorporated into ongoing preparation. Access to comprehensive manuals and resources from other Recovery Colleges was also noted as important for guiding peer educators, providing them with practical frameworks to support their roles.

In addition to support, fostering self-reflection and personal growth in peer educators were identified as crucial aspects of empowerment. Participants believed encouraging peer educators to engage in self-growth not only enables them to grow personally but also enhances their ability to foster similar growth in the participants they work with. Alice, a peer educator, explained, *“It’s empowering because it’s not just belonging to the community but being a part of developing and creating that community,”* highlighting how empowerment extends beyond individual growth to contributing meaningfully to the Recovery College environment.

Training and education were seen as foundational to empowerment as well. Participants emphasized that when peer educators receive both theoretical and hands-on training, they feel more confident and competent in delivering high-quality programs. Providing peer educators with adequate resources and training opportunities equips them with the knowledge and skills they need to succeed.

#### 3.2.5 Implementation factors

This theme captures how the outcomes of co-production are shaped by institutional and structural factors, particularly those that affect the recognition and sustainability of peer educator roles within Recovery Colleges. Participants identified two main sub-themes: equitable funding, and roles and responsibilities.

A recurring concern among participants was the inconsistent and sometimes inadequate compensation of peer educators, which was viewed as undermining the foundational principles of equality and mutual respect in co-production. Equitable pay is not only a matter of fairness but also essential for validating the professional contribution of lived experience. Kevin, the program organizer, stated:

*“We talk about peer support, and people meant to be equal and empathetic. But if you’re not in a paid role and people are attending, there’s a power imbalance. So, things about understanding what it means to have a power imbalance when there’s a pay difference…”*

This quote reflects the broader tension between peer educator roles being perceived as informal or voluntary versus recognizing them as legitimate professional positions, deserving of structured payment. Addressing these disparities is fundamental to preventing hierarchies and fostering truly collaborative spaces within Recovery Colleges.

Regarding roles and responsibilities, participants stressed the importance of clarity and stability in defining the roles and responsibilities of peer educators. They believed that clear guidelines help peer educators understand their roles within the Recovery College framework, while stability in these roles is necessary to maintain consistency in the quality of service provided. Participants also emphasized the need for guidelines that outline the possibility and process for peer educators to propose new courses in Recovery Colleges. Providing such guidelines would encourage innovation and give peer educators the opportunity to contribute meaningfully to program development, ensuring that courses remain relevant and responsive to community needs.

### 3.3 Practical recommendations by theme

**Table 2** summarizes actionable recommendations based on these themes to sustain and enhance peer educator involvement in Recovery Colleges.

## 4 Discussion

This study provides insights into the ongoing involvement of peer educators in Recovery Colleges, highlighting key factors that influence their engagement, co-production efforts, and the success of these programs. In addressing its two central objectives, identifying facilitators and barriers to peer educator engagement and co-developing strategies to sustain this involvement, the study builds on previous research arguing that sustainable change in community mental health requires participatory frameworks that act as vehicles for empowerment, enabling individuals to engage meaningfully in their communities (32). These insights align with the foundational values of Recovery Colleges, where co-production, inclusion, and peer leadership serve as core mechanisms for change. The present study contributes to this perspective by examining how these principles are operationalized through the role of peer educators in Canadian Recovery Colleges, and what structural conditions support or constrain their engagement. As a participatory research initiative, this study stands out for being co-designed by the advisory peer educators, peer educators who took on additional responsibilities as members of the peer advisory committee, for the benefit of their fellow peer educators. This approach not only enhances the relevance of the findings but also underscores the value of inclusive and collaborative methodologies in mental health research.

Our findings, guided by collaborative process rooted in curiosity, commitment, communication, compassion and capability, build upon and enrich existing literature by highlighting the centrality of inclusivity, connectedness, adaptability, empowerment, and implementation factors in fostering effective peer involvement in Recovery Colleges. While previous studies on Recovery Colleges often focus on the experiences of students and clinical staff (20, 24), this study places peer educators at the center of analysis and offers insights into co-production dynamics, particularly regarding recruitment, training, and workplace support. Although it includes perspectives from program organizers, these are used to contextualize and deepen the understanding of peer educator experiences, with particular attention to the value of lived experience. This emphasis on co-production and shared leadership resonates with Shanks et al.’s (13) calls for non-hierarchical models in recovery-oriented practices, adding depth to how these principles are operationalized.

The theme of Inclusivity mirrors findings from Moroz et al. (10), who emphasized the importance of equitable and diverse participation in mental health programs. Participants in this study further emphasized the value of inclusive and equitable practices, aligning with prior research that highlights non-judgmental, open, and accessible approaches in Recovery Colleges (15, 20, 39). This study extends previous work by illustrating how the active recruitment of peer educators with diverse lived experiences, cultural identities, and educational or professional backgrounds enriches Recovery College environments and promotes inclusivity. Moreover, it highlights challenges such as unequal access and technical barriers faced by peer educators and proposes actionable strategies, including diverse outreach and rotational leadership roles, to address these issues.

Connectedness, a vital theme in our study, complements existing research that highlights the importance of social support in mental health recovery (14). Our findings extend this literature by emphasizing the importance of fostering peer-to-peer connectedness among peer educators through structured peer support, collaboration, and community-building efforts. Also, this study advocates for fostering collaboration not only within Recovery College teams but also across Recovery College centres, encouraging province-wide and national communities of practice to enhance learning and resource-sharing.

The identification of Organizational Adaptability as a key theme adds nuance to the existing literature on Recovery Colleges by emphasizing the necessity of “failing forward,” or learning from setbacks, in fostering growth and innovation. This underscores the dynamic nature of co-production, emphasizing that Recovery Colleges must remain responsive to evolving community needs and feedback. This finding complements prior literature emphasizing flexibility as a foundational element of Recovery College co-design’s success (12, 15, 24).

Finally, the themes of Empowerment and Implementation Factors provide practical insights into supporting peer educators. From offering comprehensive training to addressing equitable compensation, these findings address gaps noted in previous studies, such as Dalgarno & Oates (25), who identified challenges related to burnout and role clarity among peer educators. This study highlights how empowerment, facilitated by clear roles, robust training, and equitable workplace practices, enhances peer educators’ capacity to foster growth and recovery among participants. This aligns with the Recovery College principle of valuing lived experience as expertise (23).

While this study aimed to provide practical suggestions for sustaining the involvement of peer educators in Recovery Colleges, we acknowledge that empowerment, co-production, and implementation are complex, evolving concepts. Our findings offer a perspective based on contextualized experiences of peer educators in Recovery Colleges, yet we echo previous research in recognizing that transformative change in community mental health requires long-standing commitments, critical reflection, and strategic research and action (32). As stated by Ornelas and colleagues, on-going opportunities to participate meaningfully in community contexts are essential for achieving personal empowerment and lasting community integration (32). Further, we recognize that how Recovery Colleges are operationalized varies considerably based on institutional contexts, resource availability, and differing interpretations of core concepts such as co-production (40). Further research is needed to explore how Recovery Colleges can best balance contextual flexibility to recovery-oriented principles, especially regarding peer educator leadership, structural supports, and the transformative use of lived experience.

## 5 Limitations

This study has some limitations. First, the relatively small number of participants in the study may limit the generalizability of the findings. Second, the focus on Canadian Recovery Colleges may not fully capture the diversity of Recovery College experiences globally. Additionally, to prioritize building trust and creating a comfortable environment for participants, we chose not to gather detailed demographic information, which resulted in a lack of comprehensive participant descriptions. Finally, while the participatory approach enhanced the relevance of the study, the involvement of the advisory peer educators in validating themes and selecting illustrative quotes may have influenced the interpretation of findings. This potential limitation reflects the collaborative nature of participatory research, where co-analysis can introduce particular perspectives. However, this influence is balanced by the value participatory methods bring in ensuring the analysis remains grounded in lived experience.

## 6 Conclusion

This study provides insights into sustaining peer educator engagement in Recovery Colleges by identifying key factors and offering actionable recommendations to improve recruitment, training, and workplace support, based on peer educator and program organizer experiences. Key recommendations to support peer educators in Recovery Colleges are offered based on five main themes: Inclusivity, Connectedness, Organizational Adaptability, Empowerment, and Implementation Factors. The Participatory Action Research approach of this study, leveraging the enthusiasm, wisdom and knowledge of the study advisory peer educators, not only enriched the findings but also reinforced the importance of inclusive and collaborative methodologies in mental health research.

## Data Availability

The raw data supporting the conclusions of this article will be made available by the authors, without undue reservation.
