# Supplementary material for: “We’re all learning together”: exploring peer educator engagement in Recovery Colleges through a participatory research approach
Source: Front Psychiatry. 2025 Jul 7;16:1601408. doi: 10.3389/fpsyt.2025.1601408 (PMC12277248; doi:10.3389/fpsyt.2025.1601408)
Supplement: Supplementary file 1 [file DataSheet1.zip › Project Survey Questions.docx]

Supplementary Material 1

# Supplementary Material (Survey)

Recovery College Research Project Survey

This survey is part of a research project: Learning from the Experiences of Recovery College Peer Educators and Program Organizers to Inform the Implementation of a Transform Substance Use Care at Vancouver Coastal Health. Thank you for participating in our survey! Please look over the following consent form before proceeding. H22-03124 Research Study Consent Form V.3 March 31 2023 Edited.PDF

I consent to participating in this research project:

- Yes; please print your name: __________________________________________________

I consent to being contacted by the research team for purposes of learning about study results.

- Yes
- No

This survey will take 10-15 minutes to complete and participation is completely voluntary.

What province or territory is your Recovery College program located in?

- Alberta
- British Columbia
- Manitoba
- New Brunswick
- Newfoundland and Labrador
- Nova Scotia
- Ontario
- Prince Edward Island
- Quebec
- Saskatchewan

What is your role in the Recovery College?

- Peer Educator
- Program Organizer

How would you describe your role?

________________________________________________________________

How long has your Recovery College been running?

- 10+ years
- 5-10 years
- 3-4 years
- 1-2 years
- less than 1 year __________________________________________________

How many peer educators work with your Recovery College?

________________________________________________________________

Display this question:

If What is your role in the Recovery College? = Peer Educator

How many years have you been a peer educator?

- 10+ years
- 5-10 years
- 3-4 years
- 1-2 years
- less than 1 year __________________________________________________

Display this question:

If What is your role in the Recovery College? = Program Organizer

How many years have you worked as a program organizer?

- 10+ years
- 5-10 years
- 3-4 years
- 1-2 years
- less than 1 year __________________________________________________

What kinds of courses does your Recovery College offer? Please check all that apply:

- Multi-session courses
- Workshop or single sessions
- Social support
- Activity-based training
- Training
- Support group
- Special events
- Other __________________________________________________

How many years have you done peer work in general?

- 10+ years
- 5-10 years
- 3-4 years
- 1-2 years
- less than 1 year __________________________________________________

Display this question:

If What is your role in the Recovery College? = Program Organizer

How many years have you worked with peers?

- 10+ years
- 5-10 years
- 3-4 years
- 1-2 years
- less than 1 year __________________________________________________

Does your organization provide training for peer educators?

- Yes
- No
- Not sure __________________________________________________

Display this question:

If Does your organization provide training for peer educators? = Yes

What does this training consist of?

________________________________________________________________

Display this question:

If Does your organization provide training for peer educators? = Yes

Are peers paid to take the training?

- Always
- Mostly
- Not paid

Display this question:

If Are peers paid to take the training? = Always

Or Are peers paid to take the training? = Mostly

Can you please tell us more about payment for facilitator training at your college?

________________________________________________________________

Did you have prior training or preparation to be a peer educator in a recovery college?

- Yes
- No __________________________________________________

Display this question:

If Did you have prior training or preparation to be a peer educator in a recovery college? = Yes

What did that consist of?

________________________________________________________________

Display this question:

If Did you have prior training or preparation to be a peer educator in a recovery college? = Yes

Did you feel well prepared as a result of this training?

- Yes
- Somewhat
- No __________________________________________________

Display this question:

If What is your role in the Recovery College? = Program Organizer

How would you describe your hiring criteria for peer educators at your Recovery College?

- Formal
- Informal

Display this question:

If How would you describe your hiring criteria for peer educators at your Recovery College? = Formal

Or How would you describe your hiring criteria for peer educators at your Recovery College? = Informal

Can you tell us more about your hiring criteria for peer facilitators?

________________________________________________________________

Does your Recovery College have dedicated staff support for the peer educators?

- Yes
- No
- Not sure __________________________________________________

Display this question:

If Does your Recovery College have dedicated staff support for the peer educators? = Yes

Can you tell us more about that?

________________________________________________________________

Over the years has your Recovery College made changes to how peer educators are supported in their work?

- Yes
- No
- Not sure __________________________________________________

Display this question:

If Over the years has your Recovery College made changes to how peer educators are supported in thei... = Yes

Can you please tell us more about the changes your Recovery College has made?

________________________________________________________________

Display this question:

If What is your role in the Recovery College? = Program Organizer

Are you planning any changes to how you prepare the peer educators in your program?

- Yes
- Maybe
- No

Display this question:

If Are you planning any changes to how you prepare the peer educators in your program? = Yes

Or Are you planning any changes to how you prepare the peer educators in your program? = Maybe

Can you please tell us more?

________________________________________________________________

What do you like about your work with the Recovery College?

________________________________________________________________

Would you recommend this work to others?

- Yes
- Maybe
- No

Can you please tell us why?

________________________________________________________________

Display this question:

If What is your role in the Recovery College? = Peer Educator

Is there anything else you'd like us to know about your experiences as a peer educator?

________________________________________________________________

Display this question:

If What is your role in the Recovery College? = Program Organizer

Is there anything else you'd like to add about supporting peer educators in your Recovery College?

________________________________________________________________

Thank you for taking time completing the survey. We appreciate your time and consideration. For taking this survey, we are holding weekly draws for $25 thank you gift cards. Would you be interested in participating in the draw?

- Yes
- No

Display this question:

If Thank you for taking time completing the survey. We appreciate your time and consideration. For t... = Yes

Please entre your name and email address.

________________________________________________________________

Would you be interested in being contacted to take part in a 30-60 minutes follow-up interview later on? The interviews will give us an opportunity to ask more detailed questions about your program and your work. Participants will be offered an honorarium of $50 for their time.

- Yes
- No

Display this question:

If Would you be interested in being contacted to take part in a 30-60 minutes follow-up interview la... = Yes

Please entre your name and email address.

________________________________________________________________
