# Supplementary material for: “We’re all learning together”: exploring peer educator engagement in Recovery Colleges through a participatory research approach
Source: Front Psychiatry. 2025 Jul 7;16:1601408. doi: 10.3389/fpsyt.2025.1601408 (PMC12277248; doi:10.3389/fpsyt.2025.1601408)
Supplement: Supplementary file 1 [file DataSheet1.zip › Project Interview Guide.docx]

Supplementary Material 2

# Supplementary Data (Interview Guide)

Learning from the Experiences of Recovery College Peer Educators and Program Organizers to Inform the Implementation of a Transformative Model of Mental Health and Substance Use Care at Vancouver Coastal Health

**Purpose:**

The aim of this study is to better understand the experiences of peer educators and program organizers from Recovery Colleges across Canada to in turn help prepare local peer educators for successful involvement with the new Recovery College YVR. We plan to base new VCH Mental Health and Substance Use services peer facilitator training materials on the study findings. We hypothesize that peers engaged in Recovery College course facilitation are better equipped to manage their own wellbeing and that thoughtful preparation for these roles is key.

We want to hear about what has worked well for you and your program and what you would have done differently to set peer facilitators up for success.

Materials to be sent in advance: Consent form

**Introduction**

- Introduce self.
- Confirm purpose and structure of interview.
- Informed consent – review consent form (sent out in advance); confidentiality; recording (remind them they can ask to have the recording turned off at anytime, refuse to answer any questions they don’t want to answer or withdraw their consent at anytime during the interview). Ask them to electronically sign the consent form and to keep a copy for their records.
- Ask if they have any questions before you turn on the recording and begin the interview.
- The Zoom program will let them know when the recording has started.

**Questions**

Please tell me about your role with your Recovery College.

Can you tell me more about your experience with Recovery College?

How did you find out about the Recovery College?

Please describe your interest in this particular model of service delivery.

a. What made you want to get involved?

Please describe your history of working with/ or as a peer facilitator(s) prior to the start of your Recovery College.

a. How was that experience different to your Recovery College work?

*Peer Educator:* What have been your most positive experiences in your role as a peer educator?

a. What have been some challenges?

*Program Organizer:* What have been your most positive experiences supervising/employing peer facilitators with a recovery college?

a. What have been some challenges?

What formats of courses do you offer? (e.g., Zoom, in person, workshop, lecture, support group)

Please tell us about how co-facilitation works at your Recovery College (e.g., Peers only, peer with clinician, other)

1. What has been working well with this co-facilitation model?
2. What has been working less well? How have you tried to address this?
3. Prompt about crisis management support. Did you feel supported? What would have helped?

What resources (human, philosophical, practical, and organizational) did you find to be helpful to supporting the peer facilitation aspect of the Recovery College?

What aspects of your location, or socio-political context are relevant to understanding how the Recovery College co-facilitation worked in your setting? i.e. social determinants of health

What are your recommendations for recruiting, training and supporting Recovery College peer facilitators?

What are the key learnings, or take-away messages, you can share about your experiences with the Recovery College?

I’ve finished my questions. Are there any other relevant points you’d like to add?

**Closure**

- Turn off the recording
- Thank them for participating
- Let them know next steps
